# Pulmonary arteriovenous malformation exhibiting recanalization >10 years after coil embolization: Two case reports

**DOI:** 10.1097/MD.0000000000018694

**Published:** 2020-01-10

**Authors:** Shun Takao, Takeshi Masuda, Takahiro Yamada, Kakuhiro Yamaguchi, Shinjiro Sakamoto, Hayato Matsushima, Yasushi Horimasu, Taku Nakashima, Shintaro Miyamoto, Hiroshi Iwamoto, Kazunori Fujitaka, Hironobu Hamada, Noboru Hattori

**Affiliations:** a Department of Respiratory Internal Medicine, Hiroshima University Hospital, Minami-ku; b Department of Neurology, Suiseikai Kajikawa Hospital, Naka-ku, Hiroshima, Japan.

**Keywords:** coil embolization, long-term follow-up, pulmonary arteriovenous malformation, recanalization

## Abstract

**Rationale:**

Some patients with pulmonary arteriovenous malformation (PAVM) present with hypoxemia and life-threatening complications, including stroke and cerebral abscess. Catheter embolization is currently the preferred treatment for PAVM. However, previous studies have revealed that the incidence of PAVM recanalization is approximately 10% 5 to 7 years after embolization. In contrast, there are no studies where recanalization has occurred over 10 years after embolization.

**Patient concerns:**

Herein, we report 2 cases diagnosed with cerebral embolism due to PAVM recanalization 13 years and 30 years after catheter treatment, in case I and II, respectively.

**Diagnoses:**

Both cases were diagnosed with PAVM recanalization on chest computed tomography (CT) examination performed after cerebral embolism development. Furthermore, pulmonary artery angiography revealed blood flow from the pulmonary artery to the vein in the PAVM, confirming PAVM recanalization.

**Interventions:**

Coil re-embolization was performed for the all recanalized PAVM.

**Outcomes:**

All the target lesions were successfully re-embolized in both cases. However, in case I, the second recanalization of embolized PAVM was confirmed 1 year after coil re-embolization. Consequently, the third embolization was performed in case I. In contrast to case I, the patient in case II was followed up without recanalization for 2 years after embolization.

**Lessons:**

We described the first 2 cases diagnosed with PAVM recanalization >10 years after the first catheter embolization. These cases suggest that patients with PAVMs should undergo life-long follow-up after catheter embolization.

## 1 Introduction

Pulmonary arteriovenous malformation (PAVM) is an abnormal direct connection between the pulmonary arteries and veins through a thin-walled aneurysmal sac, resulting in an intrapulmonary right to left shunt.^[1,2]^ Patients with single or small PAVM may be asymptomatic. However, bloody sputum, dyspnea, or cyanosis may be observed in patients with large or multiple PAVMs. In addition, approximately 30% of patients with PAVMs experience severe complications such as cerebral abscess, cerebral infarction due to bacteria, or emboli that bypass the pulmonary capillary bed.^[3]^

The preferred treatment methods for PAVM include catheter embolization and surgical resection. Of these, catheter embolization is considered the ideal choice, as it is associated with reduced loss of lung function and is less invasive than surgical resection.^[4–6]^ However, previous studies have reported that embolized PAVMs can recanalize after the first embolization, developing complications including transient ischemic attack and cerebral emboli due to recanalization.^[4]^ Therefore, periodic follow-up is recommended after catheter embolization.^[7,8]^

However, to the best of our knowledge, no studies have investigated the incidence of PAVM recanalization >10 years after the first catheter embolization. Herein, we report 2 cases of PAVM recanalization occurring 13 and 30 years after catheter treatment in case I and II respectively, in which both patients presented with cerebral emboli.

## 2 Case report

### 2.1 Case I

**Figure 1:**
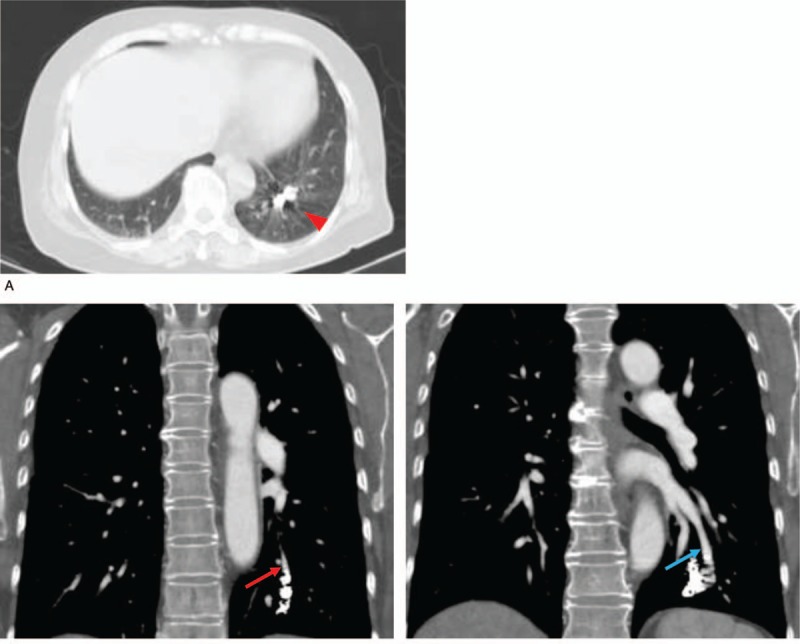
(A) Computed tomography (CT) revealed that the coil had been placed in the lower lobe S10 of the left lung in 2004 (arrow head). (B) Contrast CT revealed a contrast effect in the inflow (red arrow) and outflow (blue arrow) blood vessels of the pulmonary arteriovenous malformation (PAVM) in the lower lobe S10 of the left lung.

A 65-year-old woman without smoking history developed a brain abscess, and computed tomography (CT) revealed a PAVM in the left lower lobe of the lung in December 2003. In June 2004, coil embolization was performed for PAVM, although no follow-up was performed. In June 2017, she was diagnosed with aphasia and right frontal cortical cerebral infarction based on head magnetic resonance imaging (MRI) findings. In addition, contrast CT revealed shunt flow in the previously embolized PAVM (Fig. 1). Thus, she was diagnosed with cerebral embolism due to PAVM recanalization.

**Figure 2:**
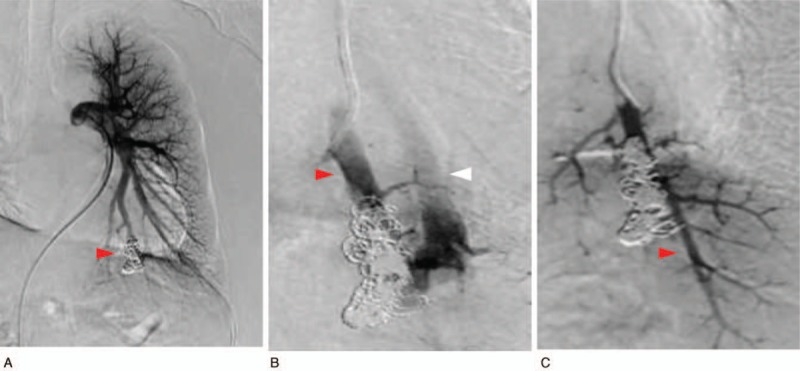
Angiography showed the embolus coil in the lower lobe S10 of the left lung (A: arrow head), and revealed blood flow from the posterior left lower lung artery (B: red arrow head) to the vein (B: white arrow head) of the PAVM (A). Delay in the flow timing of the pulmonary vein and a new, normal pulmonary artery branch were observed following embolization (C: red arrow head).

Pulmonary artery angiography revealed blood flow from the pulmonary artery to the vein in the PAVM in the lower lobe S10 of the left lung (Fig. 2). This recanalized PAVM was a simple type with one feeding artery flowing from the posterior left lower lung artery (A10). Subsequently, coil re-embolization was performed for the recanalized PAVM in the proximal end of the original coil with detachable hydrogel-coated coils and follow-up was performed using CT. Non-contrast CT was performed half a year after re-embolization, confirming PAVM sac shrinkage. However, 1 year after coil re-embolization, follow-up transesophageal echocardiography was performed and right to left shunt was detected. Therefore, contrast CT was also performed, revealing the second recanalization of embolized PAVM. Consequently, the third embolization was performed in August 2018. The patient was followed up without recanalization for 1 year after the third embolization.

Separately, she had frequent episodes of nose bleeding and telangiectasia in the upper gastric corpus, tongue, and intranasal mucosa. Additionally, her father had frequent episodes of nasal bleeding. Based on these observations, she was diagnosed with hereditary hemorrhagic telangiectasia (HHT) in accordance with Curacao criteria.^[9]^

### 2.2 Case II

**Figure 3:**
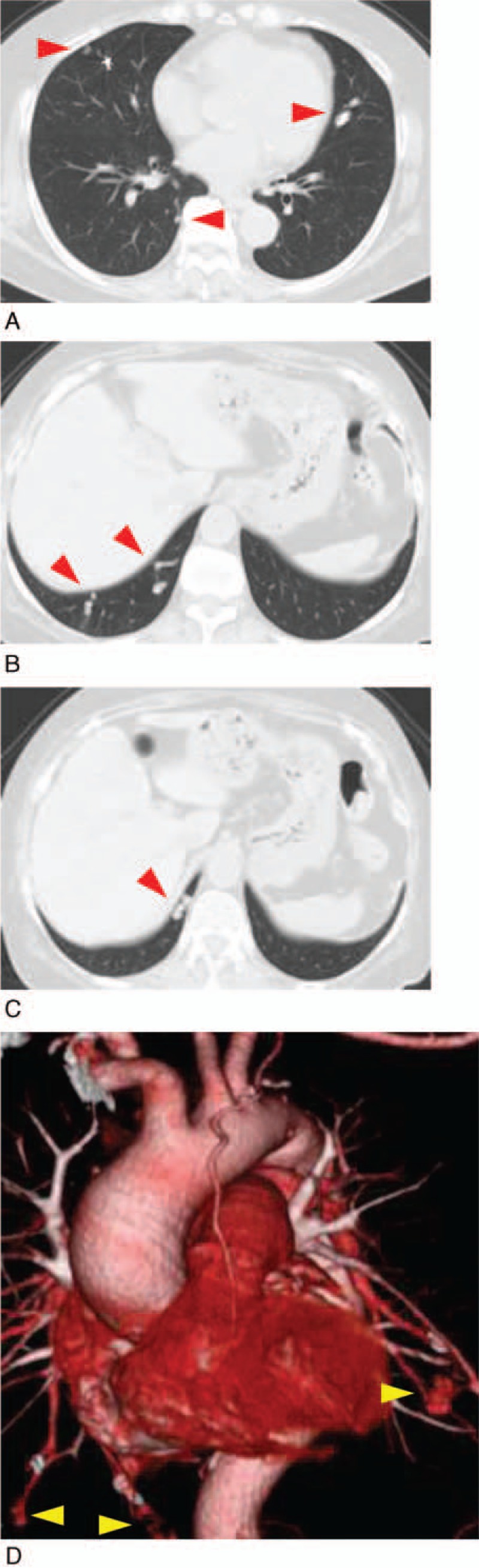
Chest computed tomography (CT) revealed multiple pulmonary arteriovenous malformations (PAVMs) in the middle lobe S4 of the right lung, lower lobe S6, S9, and S10 of the right lung, and upper lobe S4 of the left lung (A–C: red arrow heads). Three-dimensional reconstruction multidetector CT revealed multiple PAVMs (D: yellow arrow heads).

**Figure 4:**
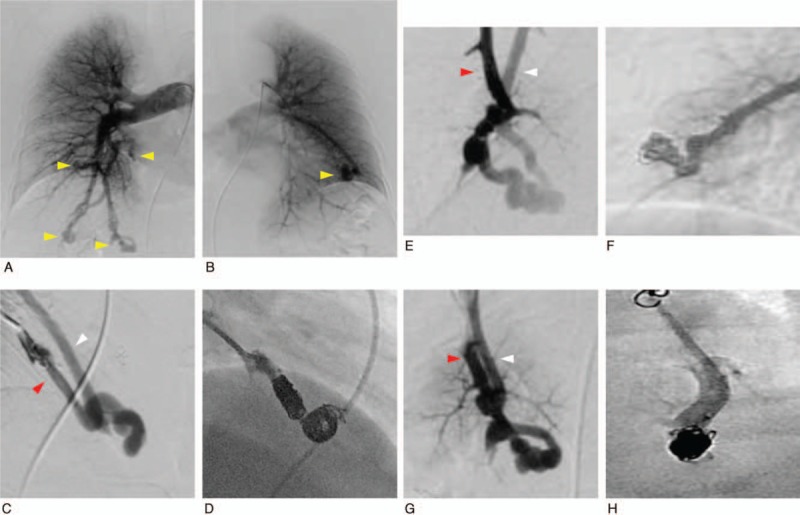
Angiography showed multiple PAVMs (A, B: yellow arrow heads) and revealed blood flow from the pulmonary artery (red arrow) to the vein (white arrow) of the pulmonary arteriovenous malformation (PAVM) in the upper lobe S4 of the left lung (C), middle lobe S4 of the right lung (E), and lower lobe S10 of the right lung (G). Blood flow from the pulmonary artery to vein disappeared after coil embolization in the upper lobe of the left lung (D), middle lobe of the right lung (F), and lower lobe S10 of the right lung (H).

A 75-year-old woman without smoking history developed cerebral infarction and underwent coil embolization for multiple PAVMs in 1987, although no follow-up was performed. In August 2017, she presented to a general neurosurgical hospital with left hemiparesis and 50% blindness. She was diagnosed with right occipital cortical cerebral infarction based on head CT findings. Furthermore, contrast CT revealed shunt flow in the previously embolized PAVMs (Fig. 3). Therefore, she was diagnosed with cerebral embolism due to PAVM recanalization. Pulmonary artery angiography revealed that all embolized PAVMs exhibited blood flow from the pulmonary artery to the vein, confirming recanalization (Fig. 4A–E). All recanalized PAVMs were simple type with a feeding artery flowing from one segmental pulmonary artery. Specifically, PAVMs in the upper lobe of the left lung and middle lobe of the right lung had flow from left superior lingular segmental artery (A4) and the right lateral segmental artery (A4), respectively. In addition, the superior segmental artery (A6), lateral basal segmental artery (A9), and posterior basal segmental artery (A10) were feeding arteries to the 3 PAVMs in lower lobe of the right lung. After recanalization was confirmed, coil re-embolization was performed in the distal end of the original coil with detachable hydrogel-coated coils for all lesions (Fig. 4F–H). Half a year after the coil re-embolization, non-contrast CT revealed that the PAVM sac was shrunk and had disappeared, confirming the success of re-embolization. The patient was followed up without recanalization for 2 years after re-embolization. In contrast to case I, this patient was not diagnosed with HHT.

## 3 Discussion

In the present report, we described 2 cases of PAVM recanalization occurring 13 and 30 years after the first coil embolization, respectively.

Catheter embolization is regarded as the gold standard treatment for PAVM due to its low incidence of complications, and because it is less invasive than surgical resection.^[9,10]^ However, PAVM recanalization can result in serious complications, as observed in the present cases. Previous studies have reported that PAVM recanalization after catheter embolization occurs in 4% to 20% of patients after the first year of follow-up.^[7,10,11]^ Furthermore, the incidence of PAVM recanalization is approximately 10% 5 to 7 years after embolization (Table 1).^[4,12,13]^ However, to the best of our knowledge, no studies have investigated the incidence of PAVM recanalization >10 years after catheter embolization. The present report is the first to describe recanalization of catheter-embolized PAVMs >10 years after embolization, as well as the cerebral embolic complications in these cases.

**Table 1:**
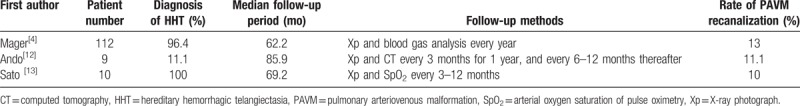
Cases of PAVM recanalization more than 5 years after embolization.

In case I, the patient experienced complications associated with HHT. Most PAVMs are congenital, and approximately 15% of Japanese patients with PAVMs have HHT, which is a heterogenous, autosomal dominant vascular disorder characterized by recurrent epistaxis, mucocutaneous telangiectasia, and visceral involvement. Conversely, approximately 50% of patients with HHT in Japan have PAVMs.^[14,15]^ In contrast, in the West, 70% of PAVM cases are complicated by HHT, and 15% to 50% of patients with HHT have PAVMs.^[9,16,17]^ Previous studies have reported that the size of PAVM increases during the natural course of the disease in patients with HHT.^[16,18]^ In other previous studies regarding the incidence of PAVM recanalization, most of the included patients had HHT.^[4,7,10,11,13]^ Therefore, the incidence of PAVM recanalization in patients without HHT remains to be clarified. In case II, we described a case of PAVM recanalization in a patient without HHT, and our results suggested that all patients should undergo long-term follow-up after PAVM catheter embolization, regardless of HHT diagnosis.

The most common cause for PAVM recanalization is blood flow through a previously placed coil nest; this applies for both initial embolization and repeat embolization.^[11,19]^ This mechanism is presumed to be caused by the fact that the pulmonary artery can stretch more than the systemic artery.^[20]^ In addition, other causes of the recanalization include pulmonary artery-to-pulmonary artery reperfusion, in which the embolized feeding artery remains occluded but small feeders develop from adjacent normal pulmonary arteries; incomplete initial treatment, in which previously untreated feeders of a complex PAVM are present; and systemic artery-to-pulmonary artery reperfusion, in which PAVMs persist due to the development of systemic arterial feeders.^[5,11]^ In both our cases, CT revealed the causes for recanalization were blood flow through the initial placed coil nest. From this observation, the cause of recanalization so long after coil embolization would likely be gradual embolized pulmonary artery expansion over years.

In case I, the PAVM developed recanalization after re-embolization, while the PAVM in case II passed without recanalization after re-embolization. This discrepancy of outcome may be due to the difference in the position of the placed coil for re-embolization. Coil re-embolization was performed in the proximal end of the original coil in case I, while it was performed in the distal end of the original coil in case II. It has been reported that recanalization treated with the coils placed distal to the recanalized coils shows better positive results than those placed proximal to the recanalized coils.^[5]^ In addition, the patient in case I was diagnosed with HHT, while the patient in case II was not. The incidence of PAVM enlargement has been shown to be more common in patients with HHT versus those without.^[21]^ Thus, having HHT may also be one of the causes of PAVM recanalization in case I.

Several methods have been utilized to evaluate PAVM recanalization after catheter embolization, including chest x-ray and CT, chest MRI, angiography, transthoracic echocardiography, and blood gas analysis. Among these, non-contrast chest CT and MRI are most frequently utilized. CT can be used to evaluate whether PAVM recanalization has occurred when the embolus is placed before the PAVM sac, and when the embolus and sac are not located within the same CT section. The success of embolization can be confirmed when the sac disappears or becomes scarred.^[22]^ In contrast, PAVM recanalization is considered to have occurred after catheter embolization when the size of the PAVM has not shrunk on CT images.^[1]^ However, when the coil is placed in the sac or exists within the same CT section as the sac, it may be difficult to evaluate recanalization due to coil artifacts. Therefore, some authors have examined PAVM recanalization using chest MRI.^[19,23]^ In both of our cases, since the sac was located outside the coil artifact, we were able to utilize non-contrast chest CT to evaluate PAVM recanalization. Follow-up for catheter embolization is recommended at 6 months, 12 months, and every 3 to 5 years after embolization.^[7,8]^ In accordance with these recommendations, we adopted such a schedule for our patients.

There is currently no established treatment for recanalized lesions. Due to its low invasiveness, catheter embolization can be repeatedly performed on recanalized lesions. Indeed, a previous study noted that 84% of recanalized PAVMs are successfully treated via catheter re-embolization.^[5]^ However, another study reported that patients who underwent embolization for recanalized PAVMs exhibited poorer responses than those with untreated PAVMs.^[19]^ Although it is not possible to give a clear answer, the vessel where the PAVM is formed is considered to extend easily after embolization due to the high compliance of the pulmonary vasculature; this would explain why the outcome of re-embolization was poor. For PAVMs that cannot be treated via re-embolization, surgical resection should be performed.^[24]^

## Acknowledgments

Informed consent was obtained from the patients for publication of this case report and accompanying images.

## Author contributions

**Conceptualization:** Takeshi Masuda.

**Writing – original draft:** Shun Takao, Takeshi Masuda.

**Writing – review & editing:** Takeshi Masuda, Takahiro Yamada, Kakuhiro Yamaguchi, Shinjiro Sakamoto, Hayato Matsushima, Yasushi Horimasu, Taku Nakashima, Shintaro Miyamoto, Hiroshi Iwamoto, Kazunori Fujitaka, Hironobu Hamada, Noboru Hattori.
